# Factors influencing implementation of mass drug administration for lymphatic filariasis elimination: a mixed-method study in Odisha, India

**DOI:** 10.3389/fphar.2024.1297954

**Published:** 2024-02-13

**Authors:** P. Ratna, Abhinav Sinha, Sanghamitra Pati, Prakash Kumar Sahoo

**Affiliations:** ICMR-Regional Medical Research Centre, Bhubaneswar, Odisha, India

**Keywords:** lymphatic filariasis, coverage, compliance, mass drug administration, India

## Abstract

**Background:** Lymphatic filariasis (LF) persists as a public health problem in India. Despite more than ten rounds of mass drug administration (MDA), LF continues to be endemic in the Dhenkanal district of Odisha. Hence, we assessed the coverage and compliance of the MDA program and explored the factors affecting it in the Dhenkanal district.

**Methods:** An explanatory mixed-method study was conducted, wherein for the quantitative survey, 552 participants aged 2 years and above were recruited following a multistage cluster random sampling during February 2022. In-depth interviews were conducted among purposively selected key stakeholders and program implementers. Descriptive statistics were used to report coverage and compliance, along with a 95% confidence interval. Qualitative data were analyzed using a thematic approach.

**Results:** We observed coverage of 99.28% and compliance of 85.87% for MDA drugs. Supervised drug administration proved to be a major pillar in increasing compliance. There was difficulty in administering drugs in urban areas due to gated societies, the absence of individuals during the day, and the perspective toward healthcare providers. Participants reported a lack of confidence in drug distributors and a fear of side effects as major causes for non-compliance.

**Conclusion:** There is a need to strengthen MDA, especially in urban areas. An urban-specific strategy, along with surveillance, behavioral change communication, and the involvement of multi-disciplinary teams, is required.

## Introduction

Lymphatic filariasis (LF) is a vector-borne neglected tropical disease (NTD) majorly prevalent in tropical and sub-tropical regions (Davis et al., 2019a). The disease impairs the lymphatic system, causing pain, disfigurement, severe disability, social stigma, and economic hardships (Weiss, 2008; Suma et al., 2013; WHO, 2022). Lymphatic filariasis can potentially be eradicated and is the fourth most common cause of disability worldwide (Agrawal and Sashindran, 2006; Hussain et al., 2014; WHO, 2022). Evidence suggests that approximately 863 million people are at risk of being infected (WHO, 2022). The World Health Organization (WHO) introduced the Global Programme to Eliminate Lymphatic Filariasis (GPELF) in 2000 (Elimination of Lymphatic Filariasis, 2022) to eliminate LF by 2020, then by 2021, and currently, it has prolonged its target by 2030 (Addisu et al., 2019; Casulli, 2021). The key strategy of GPELF is to interrupt the transmission of infection through mass drug administration (MDA) of a single dose of diethylcarbamazine citrate (DEC) plus ivermectin and albendazole administered for 4–6 years among the eligible population (Elimination of Lymphatic Filariasis, 2022; WHO, 2022). It is estimated that to have an effective interruption of disease transmission, MDA coverage and compliance of >65% are required (Babu and Babu, 2014; Hussain et al., 2014; Davis et al., 2019b; WHO, 2019).

Lymphatic filariasis is widely prevalent in the Southeast Asian region, with India being an endemic country (Kapa and Mohamed, 2020). India contributes 42% of the global LF endemic population, despite the National Filariasis Control Program and its subsequent updated forms since 1955 (Agrawal and Sashindran, 2006; Sawers and Stillwaggon, 2020). Efforts are being made by the nation to eliminate the disease from endemic pockets, with MDA as the key strategy (Banerjee et al., 2019; UNRIC, 2022). MDA coverage in India increased, gradually from 72.42% in 2004 to 87.25% in 2019 (Bhatia et al., 2018; Elimination of Lymphatic Filariasis, 2022). However, the compliance rates have been relatively low in most of the endemic areas (Ramaiah et al., 2000; Babu and Satyanarayana, 2003; Babu and Kar, 2004; Bhatia et al., 2018). Odisha is notably one of India’s highly LF endemic states, where LF persists as a formidable public health challenge, with a recent study reporting the presence of filarial antigen among 13.8% of the participants in the Khordha district (Chakraborty and Bhattacharya, 2022). Although the coverage is high, the lower compliance has been a major barrier to achieving the goals for disease elimination.

Despite several rounds of MDA, the prevalence of LF continues, which calls for an urgent need to explore the factors associated with low compliance to strengthen the existing program (Roy et al., 2013; de Souza et al., 2020; Chakraborty and Bhattacharya, 2022). It has been evident from previous studies that post-MDA coverage evaluation and compliance assessment are critical factors in ensuring the success of the MDA program while analyzing the perspectives of the beneficiaries regarding the consumption and non-consumption of MDA to encourage an implicit approach in subsequent sessions (Roy et al., 2013; Krentel et al., 2016; Banerjee et al., 2019; de Souza et al., 2020; Chakraborty and Bhattacharya, 2022). The government of Odisha endeavors to eliminate the LF from the state through the MDA drive in 15 endemic districts of Odisha in two phases (New Indian Express, 2022). We conducted a cross-sectional survey in the Dhenkanal district of Odisha that was covered under the recent MDA drive in February 2022. The study is intended to assess the coverage and compliance of MDA among the eligible population and further explore the factors associated with coverage and compliance of MDA from both the provider’s and beneficiary’s perspective.

## Materials and methods

### Study design, setting, and population

We conducted an explanatory mixed-method study in the Dhenkanal district of Odisha, India. The district is divided into eight blocks with 198 gram panchayats and four urban bodies, of which five blocks were selected for the study. The included participants were aged 2 years and above as per the guidelines of MDA distribution in India; however, for assessing knowledge, we included participants aged 11 years and above. Any severely ill individuals and pregnant women were excluded from the study, as these individuals are not covered by the MDA program. Moreover, to infer the potential operational barriers in implementing MDA, we conducted in-depth interviews with program managers, drug administrators, and people who did not comply with the received drugs.

### Sample size and sampling technique

We observed a prevalence of 76% in the previous MDA round; hence, considering it with the absolute precision of 5% at a 95% confidence interval, the prerequisite sample size for the study was calculated (Researchgate, 2022). A design effect of 1.8 and a non-response rate of 10% were added, computing the minimum required sample size to be 540. The formula used to calculate the sample size was as follows: sample size *n* = [DEFF*Np(1-p)]/[(d^2^/Z^2^_1-α/2_*(N-1)+p*(1-p)] (Charan et al., 2021). Eventually, 552 study participants in total were covered for this study. We carried out a multistage cluster random sampling to achieve the final unit of observation. First of all, we selected five out of eight blocks, namely, Bhuban in the east, Kamakhyanagar in the center, Kankadahad in the north, and Hindol and Dhenkanal Sadar in the south. In the next stage, a simple random sampling method was applied to select one rural and one urban area from each block, followed by incorporating an equal number of households from each of the selected villages/wards. Hence, we selected five villages and five wards in total.

For the initial point of the study, we randomly selected one household between the central point of the village and across the area in a random direction following the Extended Program for Immunization (EPI) recommendations (Bennett et al., 2023). Successive households were selected by including every fifth household from the first household by employing direction-based systematic random sampling. Each eligible person in the household was interviewed. For qualitative interviews, we purposively selected (*n* = 6) program managers, (*n* = 10) drug administrators, and (*n* = 10) non-compliant drug recipients.

### Data collection

The required data for the study was collected between March and April 2022, the very next month of the recent MDA session, removing the chances of recall bias. We used a pre-validated questionnaire adapted from WHO-validated tools of MDA coverage for the household-level survey (WPS, 2022). Our questionnaire inquired about the socio-demographic characteristics of the participants, such as age, gender, residence, and education level. Furthermore, it inquired about the drug distribution status in that area, the consumption status, whether it was directly observed, any experienced side effects, and the reasons for non-consumption. All participants were also assessed for their knowledge regarding the disease, including data on the symptoms experienced, causes, and treatments of the disease, and if they considered themselves at risk of infection. Children below 10 years of age were not asked questions related to knowledge. However, information regarding the consumption of MDA drugs was sought for children below 10 years of age by asking their parents whether they consumed the medicines or not. All field investigators were trained to collect data uniformly.

For in-depth interviews, a detailed guide on operational-level barriers and socio-cultural barriers faced by both drug administrators and people in the community was employed. Additionally, the guide and probes included details on the facilitators and barriers in implementing MDA, awareness among the masses, the response of the drug recipients toward MDA, and what improvements could be made. Participants who did not comply with the drugs were asked about their reasons for not consuming MDA, their perceptions about the disease and medicine, and suggestions to improve the program. Face-to-face interviews were conducted by a trained qualitative researcher (a public health graduate with experience in qualitative data collection) in the local language at a solitaire place and audio-taped after seeking consent from the participants. For the children who do not have a track record of their MDA consumption, proxy information was obtained from their parents or any closely related elder in the household.

### Data analysis

The survey data were analyzed using STATA v. 17.0 (StataCorp, Texas) software, as the investigators were well-versed with it, and STATA is a widely used and accepted software program for quantitative data analysis. Descriptive statistics were calculated as frequency and proportions, along with a 95% confidence interval (CI) as the measure of uncertainty. The total percentage of the eligible population who received the drug during the February 2022 MDA session was defined as the overall coverage, whereas compliance was defined as the percentage of the population who reported drug consumption out of those who received the drug.

We employed a thematic framework approach for the analysis of qualitative data. The data were first transcribed verbatim in the local language, Odia, by listening to the audio tapes. Each interview spanned approximately 30–40 min. This was further translated into English and was read and re-read. We carried out open coding, followed by grouping the related codes by reading the data several times. Various themes emerged from the data during coding, which were then synthesized. MAXQDA software (MAXQDA Analytics Pro 2020, VERBI GmbH, Berlin) was used for coding the qualitative data, as it provides ease for selective coding. Additionally, the authors were natives of the respective areas, and hence, they had extensive familiarity with the study’s context. To ensure consistency in coding, we had regular communications and meetings with the coders and key research team members, along with an audit trail by other team members, so that there was sufficient reflection of diversion.

### Ethical considerations

This study obtained ethical clearance from the ICMR-Regional Medical Research Center, Bhubaneswar, Odisha (letter vide no: ICMR-RMRCB/IHEC-2021/51). We collected written informed consent from all the participants prior to their participation. In the case of participants between 3 and 18 years of age, consent was obtained from their parents or their guardian.

## Results

### Socio-demographic distribution

Our study had 280 (50.72%) male participants and 272 (49.28%) female participants. The age of respondents varied from 5 to 90 years, with a mean age of 39.8 ± 18.22 years. Most of the participants were between 16 and 45 years of age (Table 1). The sample comprised 48.91% rural residents and 51.09% urban residents.

**TABLE 1:** Socio-demographic characteristics of study participants.

Variable	Category	Total (N = 552) n, % (95% CI)
Age group	3–10	21, 3.80 (2.37–5.75)
	11–15	25, 4.53 (2.95–6.61)
	16–45	315, 57.07 (52.81–61.23)
	>45	191, 34.60 (30.63–38.73)
Education	No formal education	91, 16.49 (13.48–19.84)
	Primary	106, 19.20 (15.99–22.74)
	Middle	83, 15.04 (12.15–18.29)
	Secondary	129, 23.37 (19.89–27.12)
	Higher	143, 25.91 (22.29–29.77)
Area	Rural	270, 48.91 (44.66–53.16)
	Urban	282, 51.09 (46.83–55.33)

### Knowledge of lymphatic filariasis

A total of 531 (96.2%) age-eligible (11–90 years) participants were interviewed to assess their level of knowledge of LF. In total, 481 (91%) of the participants were familiar with the most common symptoms of the disease. An approximately equal proportion of the respondents were aware of disease transmission through mosquito bites (80.98%) and used mosquito nets (81.36%) (Table 2). The majority of the participants (94.54%) were aware that LF is curable through medicine. A significantly larger proportion (99.81%) of the study participants were aware that the MDA drugs were intended for the prevention of LF. Notably, 66.48% of all respondents believed that they could be at risk of infection.

**TABLE 2:** Knowledge of lymphatic filariasis in the community.

Variable	Response	Total(N = 531) n, % (95% CI)
Mode of transmission	Mosquito bite	430, 80.98 (77.37–84.23)
	Hereditary	27, 5.08 (3.37–7.31)
	Does not know	74, 13.94 (11.10–17.17)
Most common symptoms	Swelling of limbs	452, 85.12 (81.80–88.04)
	Fever and swelling	29, 5.46 (3.63–7.74)
	Does not know	50, 9.42 (7.06–12.22)
Treatment	Medicine	502, 94.54 (92.25–96.31)
	Not curable	2, 0.3890 (04–1.35)
	Does not know	27, 5.08 (3.37–7.31)
Knowledge regarding the distribution of filarial tablets for prevention	Yes	530, 99.81 (98.95–99.99)
	No	1, 0.19 (0.004–1.04)
Do you consider yourself at risk?	Yes	353, 66.48 (62.28–70.48)
	No	146, 27.50 (23.73–31.50)
	Does not know	32, 6.03 (4.15–8.40)
Use of mosquito nets	Yes	432, 81.36 (77.77–84.58)
	No	99, 18.64 (15.41–22.22)

### Coverage and compliance of MDA

The overall coverage of MDA was observed to be approximately 99.28%, whereas 85.87% of the participants consumed MDA drugs. We observed that 14.13% of the respondents did not consume the drugs. Higher coverage and compliance rate were observed among the female participants compared to their male counterparts (Table 3). A significantly higher compliance rate of 94.07% was observed among rural residents than the 78.01% seen in urban areas. The highest compliance of 90.48% was seen among participants aged 3–10 years. We observed better compliance among literate groups than among those who had no formal education. We observed that 2.72% of the participants covered under MDA experienced side effects of the drug.

**TABLE 3:** Coverage and compliance of MDA.

Variable	Category	Frequency (n)	Coverage (N = 548) n,% (95% CI)	Compliance (N = 474) n,% (95% CI)
Overall			99.28%	85.87%
**Age**	3–10	21	21, 100	19, 90.48 (69.62–98.82)
	11–15	25	24, 96 (79.64–99.89)	21, 84 (63.91–95.46)
	16–45	315	312, 99.05 (97.24–99.80)	272, 86.35 (82.05–89.94)
	>45	191	191,100	162, 84.82 (78.92–89.58)
**Sex**	Male	280	276, 98.57 (96.38–99.60)	237, 84.64 (79.87–88.65)
	Female	272	272, 100	237, 87.13 (82.56–90.87)
**Residence**	Rural	270	269, 99.63 (97.95–99.99)	254, 94.07 (90.55–96.57)
	Urban	282	279, 98.94 (96.92–99.78)	220, 78.01 (72.72–82.70)
**Education**	No formal education	91	91, 100	77, 84.62 (75.53–91.32)
	Primary	106	106,100	94, 88.68 (81.05–94.01)
	Middle	83	82, 98.80 (93.46–99.96)	72, 86.75 (77.52–93.19)
	Secondary	129	127, 98.45 (94.51–99.81)	107, 82.95 (75.32–88.99)
	Higher	143	142, 99.30 (96.16–99.98)	124, 86.71 (80.03–91.80)

**FIGURE 1:**
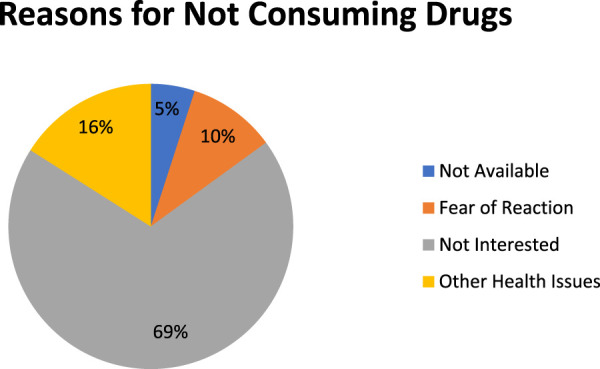
Reasons for the non-consumption of MDA drugs.

The study marked four distinct reasons for not consuming MDA among the respondents, namely, fear of side reactions (10%), having other health issues (16%), not being interested (69%), and not being available (5%) during the MDA session (Figure 1). Most of the participants (69%) reported that they were not interested in taking MDA drugs.

### Qualitative evidence

#### Facilitators and barriers in implementing MDA

The program managers felt that they encountered two major challenges in implementing MDA: 1) the unwillingness of the community members to consume the drugs and 2) the difficulties in administering drugs in urban areas.

#### Need for strengthening pre-MDA activities

A few of the program managers felt that they required more time to conduct pre-MDA activities, especially information, education, and communication (IEC) and behavior change communication (BCC) activities. This would also provide adequate time to train the drug administrators. It was commonly pointed out that appointing immediate volunteers as drug administrators was not beneficial to MDA; rather, a number of drawbacks were experienced.

“*If we have some more time for pre-MDA activities, we may achieve better results, as it will give us adequate time for preparations.*”[Program manager]

#### Challenges in implementing MDA in urban areas

The program managers mentioned that the basic urban infrastructure such as gated societies, work and lifestyle (most of the population is working and unavailable during daytime), the community’s perspective toward healthcare, and a lack of trust toward frontline workers were major barriers in implementing MDA in urban areas. Additionally, they felt that despite urban people being better educated and aware, they tend to cooperate less. Additionally, they felt that the operationalization of awareness activities was far behind in urban areas compared to rural areas. The lack of staff also played a role in low coverage and compliance in urban areas.

*“It was really strenuous to achieve coverage and compliance in urban areas compared to rural areas despite people being more aware. In urban areas, people living in buildings and apartments have security that makes it difficult for drug administrators to even enter; the longer working hours and distorted and busy schedules influence MDA coverage and are also time-consuming.* [Program manager]

*“Urban residents, although literate and aware of the disease, refused to take MDA when it comes to receiving it from ASHAs, AWWs, or volunteers, as the people, in general, stated they prefer any medication prescribed by doctors only.”* [Program implementer]

#### Facilitators in the implementation of MDA

One common response of all health workers and MDA program managers was that coverage and consumption have increased greatly this year and that the greatest strength was on-spot drug administration and supervision. The administrative-level launch of MDA in the district, involving the District Collector, Sub-divisional Officer, and Tehsildars through media, was regarded as one of the influential activities for higher compliance. Pre-MDA field visits by the district- and block-level teams of MDA planning authorities to high-LF prevalent regions were very informative and raised the enthusiasm of field-level teams. Post-MDA surveillance within an interval of 10–15 days while making efforts to cover people who did not receive MDA on the day of drug administration was also crucial. The contribution of non-governmental organizations (NGOs) such as Project Concern International (PCI) and WHO teams in the awareness drive was also beneficial in raising drug compliance. School-level awareness programs were conducted with the help of drug administrators, Scheme for Adolescent Girls (SAG) workers, NGOs, and WHO teams with the prior permission of the head of the school as well as permission of the parents through the class teachers and principal.

“*We have seen high compliance this year, which was possible due to multi-stakeholder engagement, including the government, NGOs, and WHO teams*.” [Program manager]

#### Factors associated with the non-consumption of drugs among community members

Some of the major reasons provided by people for non-consumption were not being available during the MDA; having chronic conditions such as diabetes, cardiac conditions, and psychological disorders; and taking polypharmacy. A few of the participants also reported a common cold, fever, and headache as the reasons for not taking drugs.

“I take 5–6 medicines daily. Taking additional 2–3 drugs might create a problem for me.” [Participant 6]

#### Lack of confidence in drug distributors

Some of the participants perceived that the free drugs do not work. One of the participants questioned the qualifications of the drug distributors, as they thought that the frontline workers, being not highly educated, might mislead them into taking some wrong medicines.

“*The free drugs may not work. Who knows if they are of optimal quality or not?*” [Participant 3]

#### Lack of awareness

Some of the interviewees felt that they were neither infected with LF nor was LF hereditary in their family, as they tend to keep the environment clean and take proper precautions against mosquitoes.

“*No one in our family or nearby has the disease. We do not need to consume these drugs.*” [Participant 5]

#### Fear of side effects

Most of the participants reported that they feared having side effects due to the MDA drugs. They informed that they had heard of people having side effects in the vicinity. Interestingly, none of the participants had themselves experienced or observed anyone having side effects.

“*Why should we take the drugs? If we get the side effects, who will be responsible?*” [Participant 1]

## Discussion

The study reported significantly higher MDA coverage compared to notably lower compliance. Compliance was lower among males and urban residents. We observed a higher use of mosquito nets. Multi-disciplinary teams working toward creating awareness for MDA were a strength of the program. Supervised drug distribution proved to be the major pillar in increasing compliance. However, there was difficulty in administering drugs in urban areas due to gated societies, the absence of individuals during the day due to work, and the perspective toward healthcare providers. Participants reported a lack of confidence in drug distributors and a fear of side effects as the major causes of non-compliance with drugs.

We observed the coverage and compliance rate to be 99.28% and 85.87%, respectively, which are higher than that of a recent study conducted in the Cuttack district of Odisha, which observed the coverage of MDA to be 93.2% and the compliance rate to be 73.2% (Researchgate, 2022). Evident with the mathematical prediction that 5–6 rounds of MDA with coverage of >65% can eliminate LF, and with data from previous studies, it is safe to say that our study had successful coverage and compliance (Norman et al., 2000; Plaisier et al., 2000). Nonetheless, there is a need to strengthen the program by further increasing drug compliance. We observed a lower compliance of MDA drugs among males and urban residents, which is in congruence with the results of a study conducted in four endemic districts of Odisha that reported the compliance of MDA drugs to be lower among males and in urban areas (Babu and Kar, 2004).

We observed that approximately 81% of the participants reported using mosquito nets, which is consistent with the findings of a previous study that reported the use of mosquito nets ranging from 57.9% to 90.2% in two malaria-endemic districts of Odisha (Norman et al., 2000). A probable reason for the significant use of mosquito nets in the community could be the mass awareness created by the National Vector Borne Disease Control Program (NVBDCP) in recent years regarding the use of mosquito nets in the community.

We observed that multi-disciplinary teams, including NGOs, WHO, SAG workers, and officials from the state and districts, were involved in creating awareness for MDA, which helped in strengthening pre-MDA activities such as BCC for the community and also motivated healthcare workers and drug distributors. This is similar to the findings of a previous study, which reported that involving health authorities from districts improved coverage and compliance (Babu and Kar, 2004). Future planning for MDA activities should involve higher authorities and multi-disciplinary teams. Additionally, on-spot supervised drug distribution was perceived to be the major pillar in improving compliance. This is in accordance with the international guidelines that drugs should be taken on-the-spot in the presence of drug distributors, which can easily lead to high compliance. Nonetheless, direct observation is not practiced, which leads to lower compliance. Our findings further support the practice of direct observation to improve compliance.

Program managers felt that there is a need to further strengthen MDA activities in urban areas, as gated societies with minimal access to outsiders, the unavailability of individuals due to work schedules, and a lack of trust in frontline drug distributors were challenges in operationalizing MDA activities in these areas. Similarly, a qualitative study in Odisha found that the absence of a dedicated MDA strategy for urban areas contributed to reduced coverage and compliance (Hussain et al., 2014). Urban areas have participants from varied socio-demographic and economic strata who are mostly working and unavailable during the daytime. Certain housing societies are gated colonies that do not allow entry to non-residents without proper permission. Furthermore, people have good access to healthcare facilities, including tertiary care hospitals, which makes them have less trust in frontline workers, who are mostly drug distributors for MDA. Here, making urban area-specific strategies will help in increasing coverage and compliance. Covering offices in urban areas for delivering drugs, taking resident welfare associations of gated colonies into confidence for delivering drugs, and adopting a nighttime strategy could be a few initiatives to improve MDA.

Most of the community members who did not comply with the drugs reported the simultaneous use of other medications, lack of confidence in drug distributors, lack of awareness regarding LF, and fear of side effects, which is consistent with the findings of our previous study that observed fear of side effects, not having filariasis, and taking other medications as the major reasons for the non-consumption of MDA drugs (Researchgate, 2022). Additionally, another qualitative study observed that the lack of elephantiasis cases in the locality makes people feel that the disease does not exist in their area, as people assume elephantiasis to be synonymous with LF (Hussain et al., 2014). This highlights the need for creating more awareness among the masses, which could be done by involving local community leaders and other influential actors.

### Implications for policy and practice

There is a need to improve IEC/BCC activity, specifically in the urban region. Offices and resident welfare associations could be involved in implementing MDA in urban areas. Non-consumption due to the fear of drug reactions and the unwillingness of the people to receive the drugs could be reduced through pre-MDA functional community-based awareness meetings, post-MDA revisits to follow up on any side effects of the drug, monitoring, and surveillance to identify individuals who did not receive the drug. Future studies should qualitatively explore the socio-cultural intermediaries of non-consumption of drugs. It is worth mentioning that a recent meta-synthesis protocol aims to gather qualitative evidence on facilitators and barriers in implementing MDA in India (Plaisier et al., 2000).

### Strengths and limitations

This study ensures the representativeness of the population, as participants were selected randomly. Additionally, there are very few studies that used the mixed-method study design to generate evidence on the MDA implementation program. However, this study was conducted in only one district of Odisha, which reduces its generalizability.

## Conclusion

Although we observed significant coverage and compliance of MDA drugs in the district, there is still a need for improvement, especially in urban areas. Urban area-specific strategies could help in strengthening the MDA program. Surveillance, behavioral change communication, and the involvement of multi-disciplinary teams along with influential actors are required.

## Data Availability

The raw data supporting the conclusion of this article will be made available by the authors, without undue reservation.

## Abbreviations

BCC, behavioral change communication; GPELF, Global Programme to Eliminate Lymphatic Filariasis; LF, lymphatic filariasis; MDA, mass drug administration; NTD, neglected tropical diseases; WHO, World Health Organization.
